# Research on Small Sample Multi-Target Grasping Technology Based on Transfer Learning

**DOI:** 10.3390/s23135826

**Published:** 2023-06-22

**Authors:** Bin Zhao, Chengdong Wu, Fengshan Zou, Xuejiao Zhang, Ruohuai Sun, Yang Jiang

**Affiliations:** 1 College of Information Science and Engineering, Northeastern University, Shenyang 110819, China; 2 SIASUN Robot & Automation Co., Ltd., Shenyang 110168, China; 3 Faculty of Robot Science and Engineering, Northeastern University, Shenyang 110169, China

**Keywords:** grab detection, attention mechanism, SqueezeNet, multi-object detection, deep learning

## Abstract

This article proposes a CBAM-ASPP-SqueezeNet model based on the attention mechanism and atrous spatial pyramid pooling (CBAM-ASPP) to solve the problem of robot multi-target grasping detection. Firstly, the paper establishes and expends a multi-target grasping dataset, as well as introduces and uses transfer learning to conduct network pre-training on the single-target dataset and slightly modify the model parameters using the multi-target dataset. Secondly, the SqueezeNet model is optimized and improved using the attention mechanism and atrous spatial pyramid pooling module. The paper introduces the attention mechanism network to weight the transmitted feature map in the channel and spatial dimensions. It uses a variety of parallel operations of atrous convolution with different atrous rates to increase the size of the receptive field and preserve features from different ranges. Finally, the CBAM-ASPP-SqueezeNet algorithm is verified using the self-constructed, multi-target capture dataset. When the paper introduces transfer learning, the various indicators converge after training 20 epochs. In the physical grabbing experiment conducted by Kinova and SIASUN Arm, a network grabbing success rate of 93% was achieved.

## 1. Introduction

Robot grasping technology based on depth vision has become a critical direction of current robot industry research. It is widely used in application scenarios such as assembly, grasping, and palletizing [1]. Grasping detection technology refers to the acquisition of information through visual sensors and the accurate and fast detection of the grasping frame information of the target object. Then, the robot locates the center position and the angle of the grasping target object through the grasping frame [2]. The purpose of grasping detection is to determine the appropriate grasping posture and position for the robot by grasping the visual information of the object and providing reliable perceptual information for the subsequent planning and control process. Deep visual grasping is a widely researched topic in robotics, and the methods used can be summarized as analytical and empirical [3]. In recent years, for the problem of grasp detection, research on deep learning methods using two-dimensional images as input has achieved fruitful theoretical and practical results. Research on methods related to deep visual grasping has made significant progress, as demonstrated in many kinds of literature. Satish V et al. [4] used a fully convolutional deep network to learn robot grasping strategies, which can achieve fast and reliable robot grasping of various objects. These training datasets are sampled with domain randomization using the analytical and random noise models. Using it to train a robot policy based on a fully convolutional network architecture achieved an average of 296 picks per hour. The authors of [5] provided a convenient set of training data generation methods and developed a point cloud-based deep learning method to estimate the pose of a robot’s grasping target. A point cloud simulator was introduced to generate training data, and a pose estimation algorithm based on deep learning was also designed. After training with the simulation dataset, the pose estimation algorithm can estimate the target’s pose. To solve the problem relating to optical measurement and the grasping of blanks, Wan G et al. [6] developed a matching algorithm based on deep learning and deformable templates. They designed and implemented a visual grasping strategy for industrial robots. This strategy realized the positioning recognition and grasping guidance of the object detection within the complex context of the interference of external light. The strategy was divided into two stages (object detection and object localization), and the system can accurately provide visual grasping guidance for the robot. Reference [7] proposed an end-to-end deep learning method based on point clouds to estimate the grasping pose of SCARA robots. In the PointNetRGPE model, the point cloud and class numbers are fused into a point-class vector, and several point net-like networks are used to estimate the grasping pose of the robot. More research on multi-target object grasping in academia is required, and there is no public dataset for multi-target grasping.

This article establishes multi-target grasping datasets and proposes a crawling detection method based on the attention mechanism and atrous spatial pyramid pooling (CBAM-ASPP) network model. CBAM module can focus on important information with a high weight, ignore irrelevant information with a low weight, and constantly adjust the weight so that important information can be selected in different situations. The ASPP module expands the receptive range by increasing the receptive field size without affecting the image’s resolution or adding additional computational burden. Various dilated convolutions have different convolution ranges and effectively extract multi-scale information. Because of this, the paper chose these two modules to improve the SqueezeNet network. The main contributions of this article can be summarized as follows:

(1) This article proposes a modular robot system for predicting, planning, and executing grabs and introduces camera calibration methods. It establishes a multi-object capture dataset, and preprocessing methods such as random cropping, flipping, adjusting contrast, and adding noise were used to expand the dataset;

(2) A grasping detection method based on the SqueezeNet network is proposed based on the attention mechanism and atrous spatial pyramid pooling. The paper introduces the attention mechanism network to weight the transmitted feature map in the channel and spatial dimensions. At the same time, it uses a variety of parallel operations related to atrous convolution with different atrous rates to increase the size of the receptive field and expand the receptive range. Preserving features from different ranges effectively reduces feature loss;

(3) Using the single-object dataset, the paper introduces transfer learning to pre-train CBAM-ASPP-SqueezeNet. It slightly adjusts the model parameters using the self-constructed, multi-target dataset to evaluate the CBAM-ASPP-SqueezeNet model;

(4) The CBAM-ASPP-SqueezeNet model is deployed on Kinova and SIASUN UR robots. This article evaluates the CBAM-ASPP-SqueezeNet model and successfully applies the CBAM-ASPP-SqueezeNet method to grab detection tasks on multi-objective object grab datasets. This network crawling method achieves a balance between crawling accuracy and running speed.

## 2. Overview of Robotic Arm Grasping

### 2.1. Grabbing System

**Figure 1:**
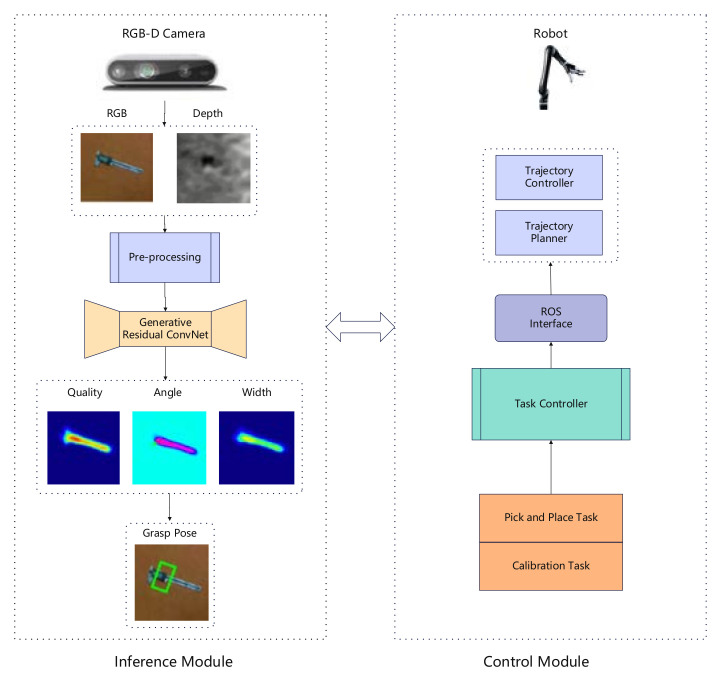
Kinova grasping system.

Figure 1 shows an overview of the proposed system’s architecture, which consists of two main modules: the prediction module for grasping target information and the robot grasping control module. (1) The grasping detection prediction module obtains scene RGB and depth images from an RGB-D camera. It infers the appropriate pose of the captured object of Kinova in the camera’s field of view. (2) The robot grabbing control module uses the grabbing information to predict the target grabbing information module, which communicates the required actions to the robot through trajectory planning and the ROS interface of the controller. Based on the network model, the grasping information is identified to control the robot to plan and adjust the position and posture of the grasping target in real time.

### 2.2. Hand–Eye Calibration

Realsense D435 Depth camera has a shooting range of up to 10 m, uses Intel’s sense SDK 2.0, and offers cross-platform support.

**Figure 2:**
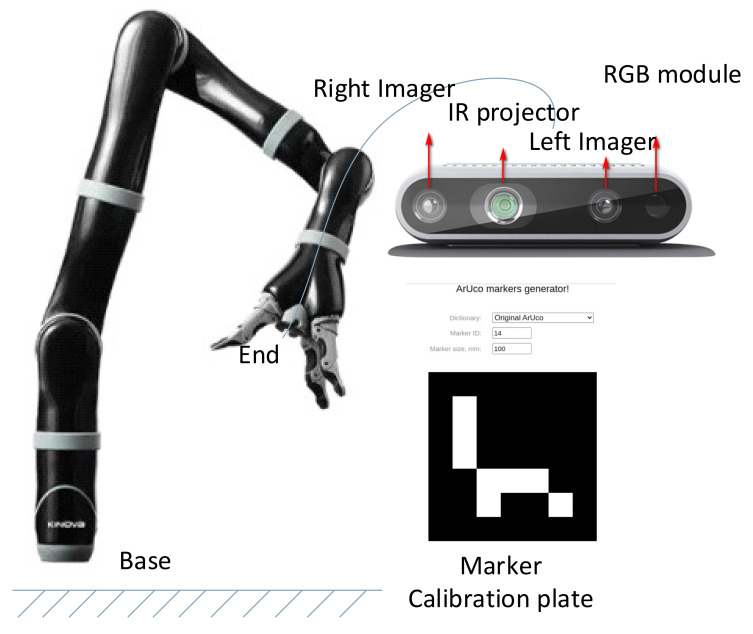
Hand–eye calibration system overview.

As shown in Figure 2, this paper uses the Kinowa robot and Realsense D435 to complete the hand–eye calibration based on the open source code of realsense-ros. The experiment adopts an automatic and non-dependent additional hardware method for eye-in-hand calibration. The calibration process is as follows:

(1) First, move the Kinowa to a suitable location by using a program or with an operator;

(2) Select the markers shown in Figure 2. The marker-based AR determines the position of the depth camera by identifying the marker’s posture in the screen. Therefore, the marker calibration plate is fixed and known in position. Once the calibration board is placed flat on the table, move the mechanical arm either manually or by script to make camera capture the calibration plate in different poses;

(3) As shown in Formula (1), each calibration detection can calculate the coordinate system of the marker calibration board. By taking 20 calibration detection samples, easy_handeye can calculate the transformation matrix TCameraEnd of the camera coordinate system relative to the end joint coordinate system. The Realsense D435 depth camera is fixed at the end of the robot, and the following formula is established:

(1) TEndBase×TCameraEnd×TMarkerCamera=TEnd_numBase×TCamera_numEnd_num×TMarkerCamera_num

The following coordinate systems are defined: The base and end are the base coordinate system and the end coordinate system of Kinova robot. The camera is the coordinate system of the Realsense D435 depth camera. Markers are part of the coordinate system of the calibration board, and the position of the calibration board is kept fixed during the calibration process. The position and posture of the camera are adjusted automatically or manually. The marker calibration plate and robot base remain unchanged during multiple sampling. The least square method is used to fit the calibration data of multiple sampling. The form of the least-square problem is $T_{f}\left(x\right)=\min\|Ax-b{\|}^{2}$; then, the optimal solution $\hat{x}$ of $T_{f}\left(x\right)$ must satisfy:

(2) $$\left\{\begin{array}{c} \partial T_{f}/\partial x_{k}\left(\hat{x}\right)=\nabla T_{f}{\left(\hat{x}\right)}_{k}=0,k=1,\cdots ,n\\ \hat{x}={\left(A^{T}A\right)}^{-1}A^{T}b=A^{†}b \end{array}\right.$$

where $T_{f}$ is least-quares equation. x^=TCameraEnd; column vectors of $A\in R_{m\times n}$ matrix are independent of each other; therefore, we obtain the above formula:

(3) $$\left\{\begin{array}{c} A^{T}A={\left(QR\right)}^{T}\left(QR\right)=R^{T}Q^{T}QR=R^{T}R\\ A^{†}={\left(A^{T}A\right)}^{-1}A^{T}={\left(R^{T}R\right)}^{-1}{\left(QR\right)}^{T}=R^{-1}Q^{T} \end{array}\right.$$

Solve $R\hat{x}=Q^{T}b$ to obtain TCameraEnd; that is, the camera is relative to the end of the conversion relationship assessed through hand–eye calibration.

**Figure 3:**
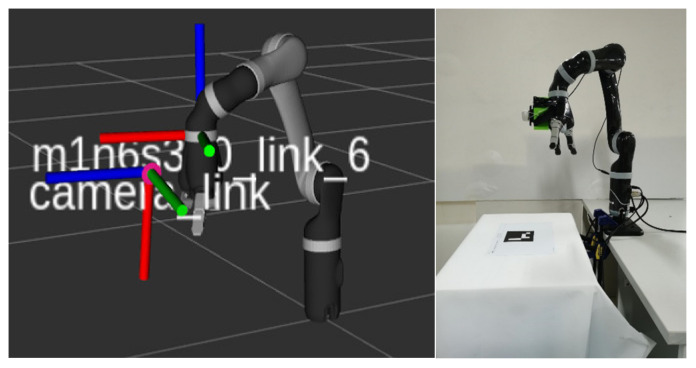
Visualization result of eye-on-hand calibration.

The hand–eye calibration results determine the position and posture representation of the camera coordinate system camera_color_optical_frame in the robot six-axis coordinate system m1n6s300_link_6. The visualization results of the hand–eye calibration results are shown in Figure 3. The left side illustrates the coordinate system of the camer_link camera and robot m1n6s300_link_6, and the right side shows the actual robot. The figure shows the calibration plate coordinate system corresponding to different sampling points, where red, green, and blue represent the X–Y–Z axes of the calibration plate coordinate system.

### 2.3. Five-Parameter Method

**Figure 4:**
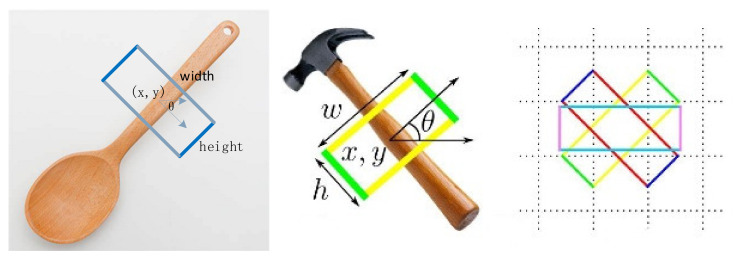
The parameters of the 5D grasp configuration.

In real scenarios, robots use five-parameter representation to grab and detect targets. This article focuses on the five-dimensional grasping representation of the two-dimensional image of the robot gripper, and the grasping posture in the two-dimensional image is shown in Figure 4.

**Figure 5:**
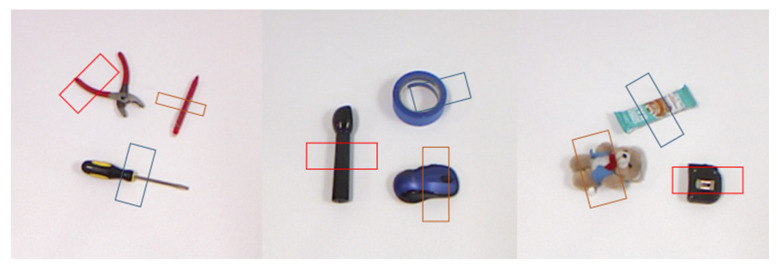
Multi-target object grasping dataset.

For the two-fingered hand grasping of the Kinova robot, where g represents a plane grasping pose. The notation (*x*, *y*) represents the position information of the end of the mechanical arm in the plane, where (*x_g_*, *y_g_*) represents the centroid of the grasping rectangle. The notation w and h represent the width and height of the parallel two-finger hand, and *h_g_* and *w_g_* represent the height and width of the rectangle of the grasping frame, respectively. Where *θ* represents the grasping angle, for the mechanical arm, it needs to rotate a certain angle in the horizontal direction to reach the grasping point; *θ_g_* is the grasping direction obtained using the angle between the grasping height hg and the horizontal axis of the image. Grab characterization is shown in Figure 5.

(4) $$\left\{\begin{array}{c} g=\left\{\theta ,x,y,w,h\right\}\\ G=\left\{x_{g},y_{g},h_{g},w_{g},\theta_{g}\right\} \end{array}\right.$$

Since the grasping angle is less sensitive than the position, the grasping angle can vary within a certain threshold, and the prediction of the grasping angle can be transformed into a classification problem. The specific conversion process is described as follows: For a parallel two-finger gripper, when grabbing an object on a plane, due to the symmetry of the gripper, the rotation angle of the gripper on the plane is $\left[0^{{}^{\circ}},180^{{}^{\circ}}\right]$; a schematic diagram of this is shown in Figure 5, and the calculated rotation angle $\theta_{cls}$ of the capture frame along the horizontal direction is shown in Formula (5), where *cls* is the classification of the angle and the range is [1, 19]. Next, 180° is divided evenly into 18 parts, and 19 represents the background class. $\theta_{cls}$ represents its corresponding angle range.

(5) $$\theta_{cls}=\left[\left(cls-1\right)\times \frac{180^{{}^{\circ}}}{18},\;cls\times \frac{180^{{}^{\circ}}}{18}\right],cls\in \left[1,18\right]$$

After data enhancement and elimination of unavailable samples, for angle prediction, the angle prediction problem is replaced with a classification problem, and 19 categories are divided into 180 degrees. Finally, each sample has an RGB image, an integer labeled from 1–19, and position parameter information (θ, x, y, w, h).

## 3. Multi-Object Grabbing Based on Lightweight Network

In the field of robotics, grasp perception is an important task. In order to solve this problem, scholars have abstracted the perception model into grasping parameter detection in a two-dimensional plane [8]. They pointed out that the feature extraction network’s influence on the algorithm’s speed is crucial. This paper studies a lightweight network model (SqueezeNet) for the problem of grasping detection. It introduces a plug-and-play network with an attention mechanism to optimize and improve the grasping Network of the SqueezeNet model. By training and testing on the single-target object dataset and the established multi-target object grasping dataset, the correctness of the CBAM-ASPP-SqueezeNet model is verified.

### 3.1. Multi-Target Dataset Production

Currently, the targets for publicly grabbing datasets are all single targets, making it necessary to create multi-target object-grabbing datasets. As shown in Figure 5, the self-constructed multi-object Cornell dataset in this article is a robot-grasping dataset used for grasping and evaluating RGB-D data. It adopts the same annotation protocol as the Cornell dataset. It can be used as a multi-object extension for the Cornell dataset. The dataset needs more sample types and quantities. Therefore, it cannot meet the standards of multi-objective, large-scale datasets. This issue can easily lead to the underfitting of the model, making it difficult for the network to learn practical features [9,10,11,12,13,14].

**Figure 6:**
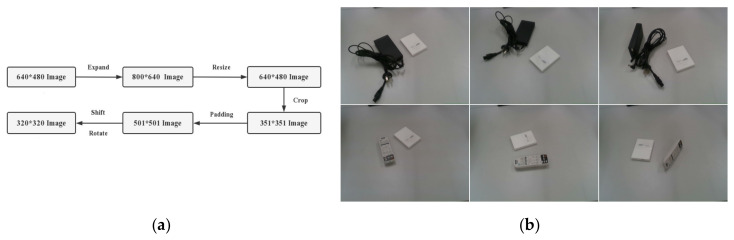
Image preprocessing extension graph: (**a**) graphic preprocessing process; (**b**) dataset extension effect.

Due to the relatively small number of multi-objective datasets, image preprocessing and data enhancement are two standard methods used to solve this problem. As shown in Figure 6a,b, data augmentation is a standard image augmentation method that can include operations such as image translation, rotation, random erasure, and adding noise to the color space. These operations can improve the diversity of image data, increase the amount of training data, reduce the risk of overfitting, improve the model’s generalization ability, and thus improve the training effect of the model.

In object detection tasks, preprocessing ensures that multiple targets do not exceed the boundary and remain in the field of view. Resize and padding operations of the image are required to eliminate object deformation and ensure that the object is in the field of view. At the same time, it is necessary to remove invalid grabbing boxes to retain the object to be grabbed. The specific operation steps are as follows: Fill the original 640 × 480 image with 80 pixels around each side to obtain an 800 × 640 image; then, resize it to restore its original size, and crop it square from the center to the surrounding area, resulting in an image size of 351 × 351. Next, expand by 75 pixels around each area, use the replicate mode in OpenCV, and increase the image size to 501 × 501. Perform several pixel translations along the horizontal and vertical directions and five random rotations within 180 °. Perform a final image resizing with a final image size of 320 × 320. These operations obtain a 100-fold enhancement relative to the original dataset.

### 3.2. The Attention Mechanism and Atrous Spatial Pyramid Pooling (CBAM-ASPP)

**Figure 7:**
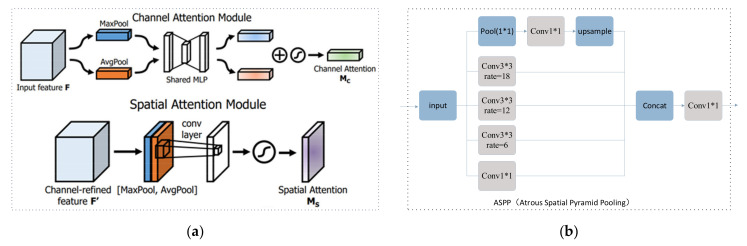
The ASPP module: (**a**) the attention mechanism; (**b**) the atrous spatial pyramid pooling.

CBAM (attention mechanism): As shown in Figure 7a, the structures of these two attention mechanisms are as follows: The attention mechanism is related to the adjusting of weights. This article can add the plug-and-play network structure CBAM to other CNN structures. CBAM utilizes both channel attention and spatial attention. This involves weighing the incoming feature maps based on channel and spatial dimensions. The larger the weight, the more meaningful the channel or spatial dimensions information is. The weights for the channels and spaces are determined in this article through MaxPool and AvgPool. 

Atrous spatial pyramid pooling (ASPP): Figure 7b shows a structure called an atrous spatial convolutional pooling pyramid module. This module expands the receptive range by increasing the receptive field size without affecting the image’s resolution or adding additional computational burden. The paper introduces a spatial pyramid pooling module with atrous convolution and upsampling to reduce feature loss. Compared with ordinary convolution, cavity convolution introduces a parameter called rate, which controls the size of the receptive field. The larger the rate, the larger the receptive field of the feature. Since targets of different scales correspond to different receptive field requirements, this paper uses three dilated convolutions with different dilation rates to operate in parallel. This obtains information on different ranges and scales around the target through upsampling operations. Different dilated convolutions have different convolution ranges, subsequently retaining features of different ranges and effectively reducing feature loss. The spatial pyramid pooling module with dilated convolution can maintain the size of the feature map and control the range of the receptive field, which helps to extract multi-scale information.

### 3.3. CBAM-ASPP-SqueezeNet

SqueezeNet has similar accuracy rates to AlexNet, and the number of parameters can be reduced by 50 times. The ReLU activation function is introduced between the compression and expansion layers. The basic module of SqueezeNet is Fire Module, and Squeezenet reduces network parameters using the following three aspects:

(1) Use a 1 × 1 convolution kernel to replace most 3 × 3 convolution kernels because the number of parameters of a 3 × 3 convolution kernel is nine times that of a 1 × 1;

(2) Reduce the number of inputs to 3 × 3 convolution kernel channels. Since the number of input channels is equal to the number of convolution kernels, the number of channels is also critical to the number of network parameters;

(3) After downsampling, in the convolutional network, each convolutional layer will generate an output feature map, which is mainly controlled by the following factors: the size of input data and the location of the downsampling layer. Larger feature maps contain richer spatial information, which can maximize classification accuracy; therefore, the downsampling layer is placed at the back. Suppose downsampling occurs in a network relatively near the front. In that case, the feature map of the overall network will shrink, which is not conducive to the performance of image classification tasks. Therefore, delayed downsampling can enrich the features in the network and improve the overall performance.

**Figure 8:**
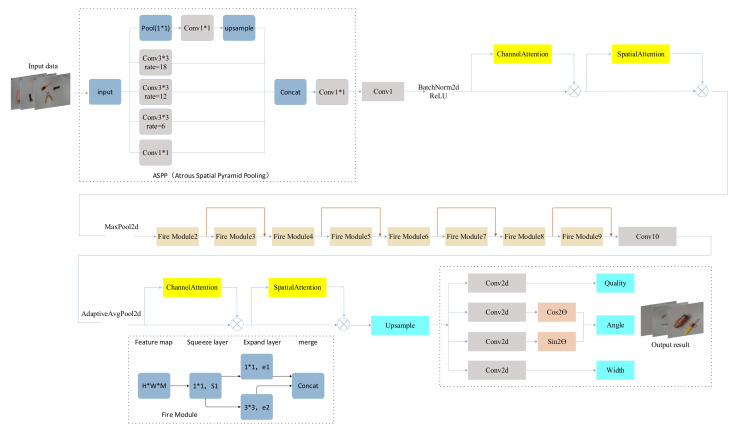
The overall structure of the proposed lightweight CBAM-ASPP-SqueezeNet model.

Figure 8 shows the overall structure of the lightweight CBAM-ASPP-SqueezeNet model. Based on the SqueezeNet network, a plug-and-play network with an attention mechanism is added to weight the transmitted feature graph in the channel and spatial dimensions. At the same time, the paper uses a variety of parallel operations of atrous convolution with different atrous rates to increase the size of the receptive field and expand the receptive range. Preserving features from different ranges effectively reduces feature loss. The grabbing network of the SqueezeNet model is optimized and improved.

The CBAM-ASPP-SqueezeNet model contains one ASPP group to increase the receptive field’s size and to preserve features from different ranges. ASPP essentially consists of a 1 × 1 convolution + pooling pyramid + ASPP Pooling composition. The CBAM-ASPP-SqueezeNet model contains two groups relating to channel attention and spatial attention to obtain channel and spatial dimension information. The first set of CBAM occurs after the first convolution, BatchNorm2d, and ReLU; the second set of CBAM is sampled before the upsample. Eight Fire Module modules increase the maximum pooling value with a step size of 2 after Fire Module 4 and Fire Module 8 are used for downsampling. The additional compositing of features was performed with the same number of channels, which are located after (Fire Module 2, Fire Module 3), (Fire Module 4, Fire Module 5), (Fire Module 6, Fire Module 7), and (Fire Module 8, Fire Module 9). Since the number of channels is different, features cannot be added directly; therefore, a 1 × 1 convolution is used to change the number of channels to achieve feature fusion. After the fusion of the feature maps, the parameters of the capture frame are finally predicted. The CBAM-ASPP-SqueezeNet output includes five dimensions corresponding to the capture parameters (x, y, w, h, θ_cls_).

### 3.4. Transfer Learning

Transfer learning is a method that uses pre-trained models or features to solve new tasks by adjusting model parameters or retraining part of the network layer. Transfer learning has a variety of applications, which can significantly improve the performance and generalizability of the model, especially when the amount of data is small [9,10,11,12,13]. In addition, transfer learning can also reduce training time and computing resource consumption because the pre-training model has learned many features, which can be directly applied to new tasks. The main idea of transfer learning is to apply the learned knowledge to new tasks to accelerate the model’s training and improve the model’s performance. It is appropriate to use transfer learning when the tasks are similar, the model parameters are weighted and biased similarly, and the input and output formats are the same.

**Figure 9:**
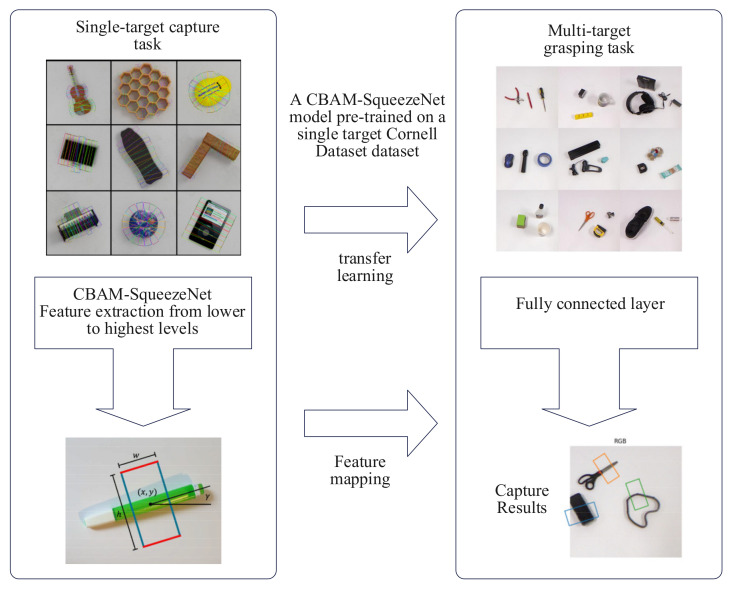
Transfer learning flow chart.

Transfer learning can be divided into three main stages: pre-training, slightly modifying, and testing. In the pre-training stage, train deep learning models are based on large-scale datasets. In the slightly modifying stage, some or all of the network layers of the pre-trained model are used to apply the model to new tasks and to perform fine-tuning on new task data. Finally, during the testing phase, the fine-tuned model is used for prediction. It should be noted that transfer learning can easily lead to overfitting when data need to be increased. Figure 9 shows the flow chart of transfer learning. This study uses transfer learning technology to solve the problem of multi-target datasets with a small amount of label data. The model pre-trained on other large datasets is used as an underlying image feature extractor, and the general image feature extraction knowledge is migrated to the multi-target capture field to reduce the dependence on label data.

Specifically, its model recognition efficiency has been reduced sparingly due to the increased complexity of the CBAM-ASPP-SqueezeNet model compared to the previous SqueezeNet. This study successfully constructed a multi-target grasping model. The CBAM-ASPP-SqueezeNet model used its pre-trained weights on the Cornell dataset to initialize the target model parameters in order to achieve the sharing of knowledge already learned by the new model. Then, the output layer is adjusted according to robot multi-objective object grasping technology requirements to slightly modify the model, using labeled image samples in the expanded multi-objective object grasping dataset.

## 4. Grab Detection Results and Analysis

### 4.1. Experimental Environment

This experimental section verifies the CBAM-ASPP-SqueezeNet model based on the public single-target Cornell and self-built, multi-target object datasets. The experimental server was used in the following environment:

1. Operating system: Ubuntu MATE16.04

2. CPU: Intel(R) Xeon(R) CPU E5-2620 v4 @ 2.10 GHz

3. GPU: Titan X Python version: 3.7.13

4. torch version: 1.10.1+cu111 torch-vision version: 0.11.2++cu111

The multi-target object grasping datasets are divided into train sets and test sets, which have been divided into folders. Because of transfer learning, the ratio of the train and test set is 7:3; the epoch is 100; the learning rate is initially set at 0.0025; batch_size is usually 1000.

The critical elements of the robot grasping system include visual perception, grasping pose estimation, trajectory planning, and grasping execution. The grabbing system platform includes robotic arms, D435 depth cameras, and laptops. The software platform includes camera acquisition, grasping position and posture estimation, grasping planning, and motion control.

### 4.2. Evaluation Indicators

This article mainly studies the multi-objective object grasping problem found in Kinova and SIASUN UR robotic arms. There are two evaluation indicators for robot grasping: point metric and moment metric. At present, moment measurement is commonly used for grasping evaluation, while grasping angle and Jaccard index standards are used to evaluate robot grasping. The CBAM-ASPP-SqueezeNet model used in this article uses rectangular metrics for evaluation.

1. The difference between the predicted capture frame angle and the effective capture frame angle is within 30;

2. The Jaccard index between the predicted capture frame $R_{p}$ (the area of the predicted capture rectangle) and the actual value $R_{t}$ (the area of the actual capture rectangle) is greater than 25%, and the Jaccard is calculated as follows:

(6) $$J\left(R_{t},R_{p}\right)=\frac{\left|\left(R_{t}\cap R_{p}\right)\right|}{\left|\left(R_{t}\cup R_{p}\right)\right|}>25\%$$

When the candidate grab box meets the above criteria, the candidate grab box is considered valid. According to this definition, the Jaccard index is equivalent to the Region of Interest (IOU) threshold determined during object detection research, which includes information on the center point of grasping and the width of the gripper opening. Since a real value rectangle can define an ample grabbable rectangular space, a predicted rectangle that overlaps 25% with the ground truth rectangle is still a good truth rectangle, as long as its direction is correct.

### 4.3. Object Grabbing and Grabbing Detection Indicators

**Figure 10:**
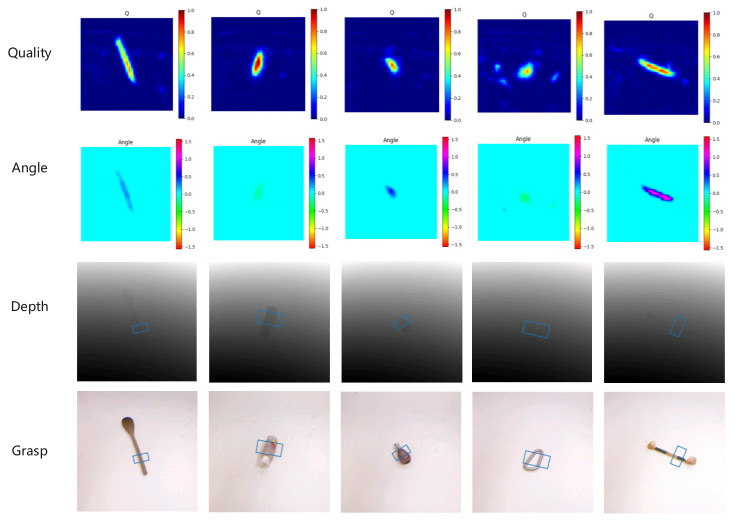
The grasping detection of the proposed model on the Cornell dataset.

The CBAM-ASPP-SqueezeNet model is pre-trained using the Cornell dataset to achieve grasping prediction. The extended version of the Cornell grab dataset includes 1035 RGB D images with a resolution of 640 × 480 pixels, including 240 positive grabbing samples of 5100 and 2909 negative grab samples of different real objects. Due to the sufficient size of the dataset, this paper pre-trains the CBAM-ASPP-SqueezeNet model. Figure 10 shows the grabbing quality, angle, and width result of the CBAM-ASPP-SqueezeNet model based on the Cornell datasets. The experimental results demonstrate that the CBAM-ASPP-SqueezeNet method can effectively predict the crawling of different types of objects.

**Figure 11:**
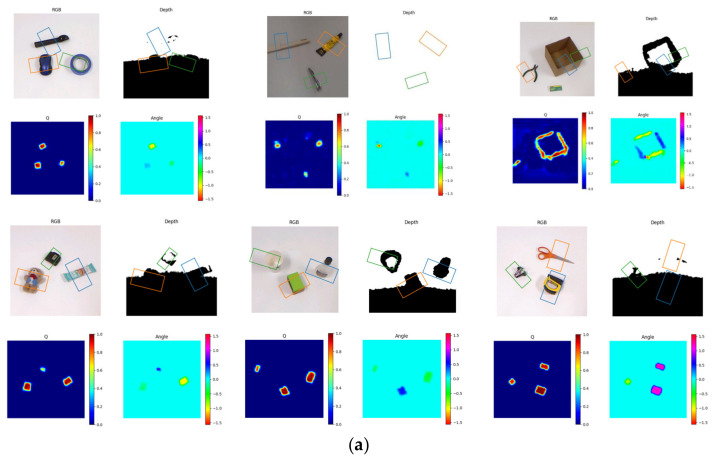

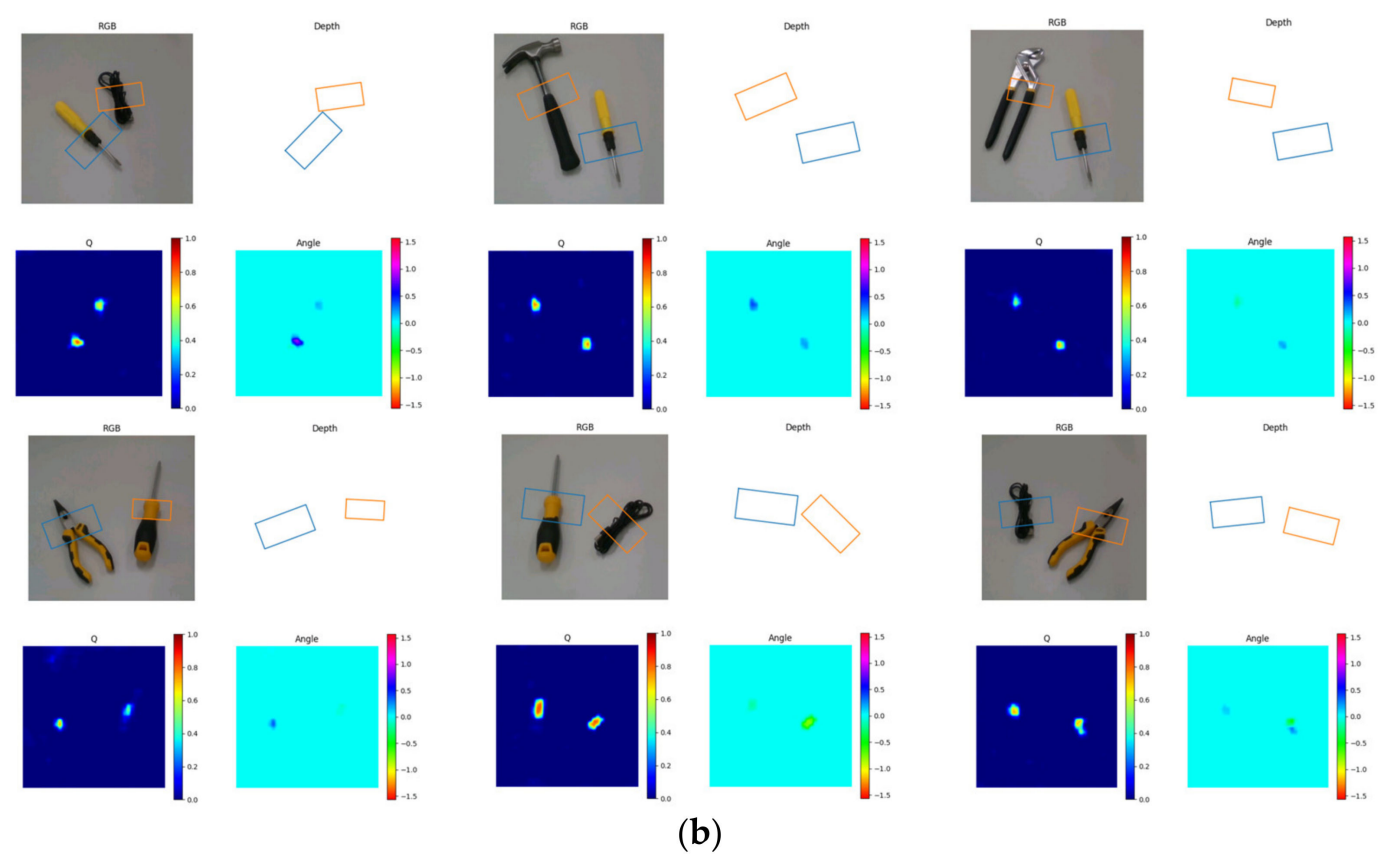
(**a**) The detection output of the proposed model using the self-built, multi-target object dataset. (**b**) The detection output of the proposed model using the self-built, multi-target object dataset.

The paper introduces transfer learning to pre-train CBAM-ASPP-SqueezeNet using a single-object Cornell dataset and slightly adjusts the model parameters using the self-constructed, multi-target object capture dataset to evaluate the CBAM-ASPP-SqueezeNet model. Figure 11a,b shows the CBAM-ASPP-SqueezeNet test results of the multi-objective objects’ grabbing effect, grabbing quality, angle, and width. Overall, the grab method based on the rectangular representation introduced in this article performs well in achieving grab prediction, and experimental results have shown its feasibility and effectiveness. This method can be applied to various object types and be widely applied in grabbing technology.

Table 1 shows the parameters of Squeeze and CBAM-ASPP-SqueezeNet, and that CBAM-ASPP-SqueezeNet has 7.5% more parameters than Squeeze.

As shown in Table 2, CBAM-ASPP-SqueezeNet has an IOU of 0.95 on the single-target Cornell capture dataset, which is a higher IOU than the one obtained for SqueezeNet.

As shown in Table 3, CBAM-ASPP-SqueezeNet has an IOU of 0.93 on the self-built multi-target object dataset, which represents a higher accuracy than SqueezeNet.

The CBAM-ASPP-SqueezeNet network, which introduces the attention mechanism and atrous spatial pyramid pooling, increases the computational complexity of the model to a certain extent. However, CBAM-ASPP-SqueezeNet is higher by about 3–5 percentage points than Squeeze on IOU, proving that the attention mechanism and atrous spatial pyramid pooling have a specific contribution to improving network performance.

**Figure 12:**
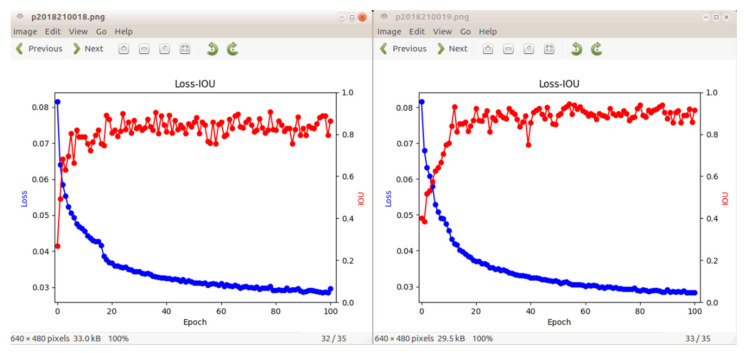
LOSS and accuracy of capture detection based on attention mechanism.

It can be seen from Figure 12 that the effect diagram of the IOU and LOSS of the capture detection based on the attention mechanism and atrous spatial pyramid pooling verifies that transfer learning and the attention mechanism have the function of accelerating the model convergence speed. At approximately 20 epochs, the CBAM-ASPP-SqueezeNet model approached the highest IOU. The left figure shows CBAM-SqueezeNet and the right figure shows CBAM-ASPP-SqueezeNet. The figure shows that CBAM-ASPP-SqueezeNet has a higher IOU than CBAM-SqueezeNet, and the model’s IOU, LOSS, and convergence speed have been improved.

### 4.4. Real Object Grasping Detection

**Figure 13:**
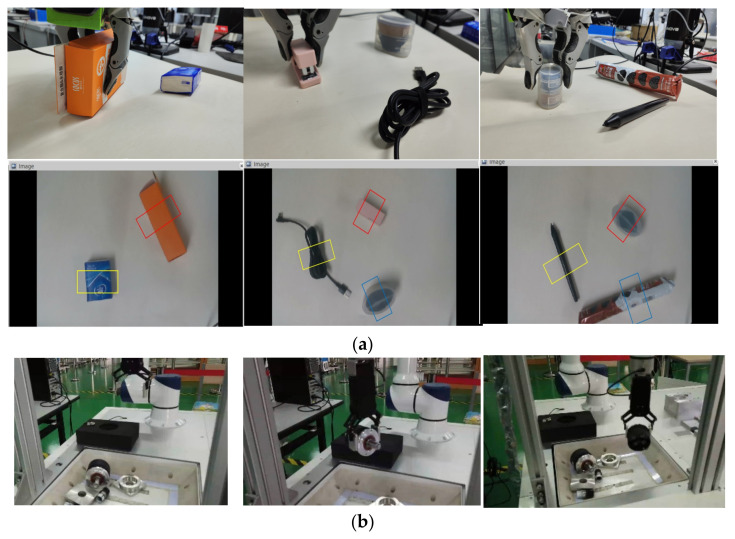
(**a**) Kinova grasping experiment for real-world robotic grasping. (**b**) SIASUN UR grasping experiment for real-world robotic grasping.

This study detected the capture of real targets. Figure 13a,b shows the grasping schematic diagrams of Kinova and SIASUN UR in a multi-object scene. This article uses the CBAM-ASPP-SqueezeNet algorithm to exhibit a fast computational speed of 15 milliseconds regarding inference speed and achieves a balance between accuracy and running speed. The method proposed in this study achieved a physical capture success rate of 93% in real object capture detection, demonstrating faster and more efficient characteristics. At the same time, during the grabbing experiment, the grabbing detection system can predict good grabbing poses, demonstrating the generalization performance of the network.

The experimental results show the widespread applicability of this target-grasping algorithm in robotic arms. This algorithm has a promising universal performance and can be easily ported to other robot systems. The CBAM-ASPP-SqueezeNet algorithm can perform well in various testing environments, providing an effective solution for achieving efficient grabbing tasks.

## 5. Conclusions

This article utilizes the attention mechanism and atrous spatial pyramid pooling module to optimize and improve the SqueezeNet model’s crawling network. Transfer learning is used to solve the problem of insufficient small sample datasets for multiple targets. The CBAM-ASPP-SqueezeNet model can be used for multiple target capture detection models. The proposed crawl detection method, whether on a single target Cornell dataset or a self-built, multi-target object dataset, has only 7.5% more parameters than the Squeeze parameter in the CBAM-ASPP-SqueezeNet model. However, the accuracy and IOU of the CBAM-ASPP-SqueezeNet network are significantly better than existing SqueezeNet crawl detection network methods, with better generalizability. Having been verified using Kinova and SIASUN crawling platforms, the CBAM-ASPP-SqueezeNet network crawling method has a reasoning speed of 315 ms, achieving a balance between crawling accuracy and running speed.

This research involved the construction of a multi-target dataset and CBAM-ASPP-SqueezeNet lightweight network model to bring several significant advantages: filling the gap in multi-objective datasets, more efficient distributed training, lower model migration costs, and easier deployment in embedded devices. The investigation also needs more research on multi-objective stacking, multi-objective sorting, and crawling, and further improvements will be made. There is still a particular gap between applying this algorithm in real and laboratory environments because there is a significant difference between unstructured and laboratory environments.

## Figures and Tables

**Table 1:** Parameters of Squeeze and CBAM-ASPP-SqueezeNet.

Network Type	Total Params	Params Size (MB)
Squeeze	209,176	0.80
CBAM-ASPP-Squeeze	226,332	0.86

**Table 2:** IOU and FPS of network on single-object dataset.

Network Type	IOU	FPS
Squeeze	0.90	97
CBAM-ASPP-Squeeze	0.95	65

**Table 3:** IOU and FPS of network on muti-object dataset.

Network Type	IOU	FPS
Squeeze	0.90	52
CBAM-ASPP-Squeeze	0.95	41

## Data Availability

Data available in a publicly accessible repository. The data presented in this study are openly available in https://github.com/SimonZhaoBin.

## References

[1] Zhang Y. (2021). Research on Robot Dynamic Grasping Technology Based on Perspective Transformation. Softw. Eng. Appl..

[2] Ni P., Zhang W., Zhu X., Cao Q. (2021). Learning an end-to-end spatial grasp generation and refinement algorithm from simulation. Mach. Vis. Appl..

[3] Zhao B., Wu C., Zhang X., Sun R., Jiang Y. (2023). Object grasping network technology of robot arm based on Attention Mechanism. J. Jilin Univ..

[4] Satish V., Mahler J., Goldberg K. (2019). On-Policy Dataset Synthesis for Learning Robot Grasping Policies Using Fully Convolutional Deep Networks. IEEE Robot. Autom. Lett..

[5] Wang Z., Xu Y., Xu G., Fu J., Yu J., Gu T. (2021). Simulation and deep learning on point clouds for robot grasping. Assem. Autom..

[6] Wan G., Wang G., Xing K., Fan Y., Yi T. (2021). Robot visual measurement and grasping strategy for roughcastings. Int. J. Adv. Robot. Syst..

[7] Wang Z., Xu Y., He Q., Fang Z., Xu G., Fu J. (2020). Grasping pose estimation for SCARA robot based on deep learning of point cloud. Int. J. Adv. Manuf. Technol..

[8] Kumra S., Shirin J., Ferat S. Antipodal Robotic Grasping using Generative Residual Convolutional Neural Network. Proceedings of the 2020 IEEE/RSJ International Conference on Intelligent Robots and Systems (IROS).

[9] Hossain D., Capi G. (2018). Multiobjective evolution of deep learning parameters for robot manipulator object recognition and grasping. Adv. Robot..

[10] Li M., He Z., Zhu Y., Jia L., Wang Y., Ding X., Cui Y. (2022). A Method of Grasping Detection for Kiwifruit Harvesting Robot Based on Deep Learning. Agronomy.

[11] Ku Y., Yang J., Fang H., Xiao W., Zhuang J. (2021). Deep learning of grasping detection for a robot used in sorting construction and demolition waste. J. Mater. Cycles Waste Manag..

[12] Liu N., Guo C., Liang R., Li D. (2022). Collaborative Viewpoint Adjusting and Grasping via Deep Reinforcement Learning in Clutter Scenes. Machines.

[13] Ribeiro E.G., de Queiroz Mendes R., Grassi V. (2021). Real-time deep learning approach to visual servo control and grasp detection for autonomous robotic manipulation. Robot. Auton. Syst..

[14] Zhang L., Zhang H., Yang H., Bian G., Wu W. (2019). Multi-target detection and grasping control for humanoid robot NAO. Int. J. Adapt. Control. Signal Process..

